# CRISPR/Cas9 mediated gene editing of transcription factor ACE1 for enhanced cellulase production in thermophilic fungus *Rasamsonia emersonii*

**DOI:** 10.1186/s40694-023-00165-y

**Published:** 2023-09-01

**Authors:** Varinder Singh, Yashika Raheja, Neha Basotra, Gaurav Sharma, Adrian Tsang, Bhupinder Singh Chadha

**Affiliations:** 1 Department of Microbiology, Guru Nanak Dev University, Amritsar-143005, Punjab, India; 2 Center for Structural and Functional Genomics, Concordia University, 7141 Sherbrooke Street West, Montreal, QC H4B 1R6 Canada

**Keywords:** *Rasamsonia emersonii*, CRISPR/Cas9, ACE1 transcription factor, Gene expression, Cellulases

## Abstract

**Background:**

The filamentous fungus *Rasamsonia emersonii* has immense potential to produce biorefinery relevant thermostable cellulase and hemicellulase enzymes using lignocellulosic biomass. Previously in our lab, a hyper-cellulase producing strain of *R. emersonii* was developed through classical breeding and system biology approaches. ACE1, a pivotal transcription factor in fungi, plays a crucial role in negatively regulating the expression of cellulase genes. In order to identify the role of ACE1 in cellulase production and to further improve the lignocellulolytic enzyme production in *R. emersonii*, CRISPR/Cas9 mediated disruption of *ACE1* gene was employed.

**Results:**

A gene-edited *∆ACE1* strain (GN11) was created, that showed 21.97, 20.70 and 24.63, 9.42, 18.12%, improved endoglucanase, cellobiohydrolase (CBHI), *β*-glucosidase, FPase, and xylanase, activities, respectively, as compared to parental strain M36. The transcriptional profiling showed that the expression of global regulator (*XlnR*) and different CAZymes genes including endoglucanases, cellobiohydrolase, *β*-xylosidase, xylanase, *β*-glucosidase and lytic polysaccharide mono-oxygenases (LPMOs) were significantly enhanced, suggesting critical roles of *ACE1* in negatively regulating the expression of various key genes associated with cellulase production in *R. emersonii.* Whereas, the disruption of *ACE1* significantly down-regulated the expression of *CreA* repressor gene as also evidenced by 2-deoxyglucose (2-DG) resistance phenotype exhibited by edited strain GN11 as well as appreciably higher constitutive production of cellulases in the presence of glucose and mixture of glucose and disaccharide (MGDs) both in batch and flask fed batch mode of culturing. Furthermore, *∆ACE1* strains were evaluated for the hydrolysis of biorefinery relevant steam/acid pretreated unwashed rice straw slurry (Praj Industries Ltd; 15% substrate loading rate) and were found to be significantly superior when compared to the benchmark enzymes produced by parent strain M36 and Cellic Ctec3.

**Conclusions:**

Current work uncovers the crucial role of ACE1 in regulating the expression of the various cellulase genes and carbon catabolite repression mechanism in *R. emersonii*. This study represents the first successful report of utilizing CRISPR/Cas9 genome editing technology to disrupt the *ACE1* gene in the thermophlic fungus *R. emersonii*. The improved methodologies presented in this work might be applied to other commercially important fungal strains for which genetic manipulation tools are limited.

**Supplementary Information:**

The online version contains supplementary material available at 10.1186/s40694-023-00165-y.

## Background

Filamentous fungi are a rich source of industrially important proteins, enzymes, organic acids and secondary metabolites. Among different fungal species, *Rasamsonia emersonii, Thielavia terrestris, Myceliophthora thermophila* and *Humicola insolens* have been widely used for the production of thermostable carbohydrate-active enzymes (CAZymes) which are involved in degradation of lignocellulosic biomass and possess various industrial application [1–3]. *Trichoderma reesei* strains have been extensively developed as a source of lignocellulolytic enzymes by industries throughout history. However, these enzymes exhibit temperature optima that falls within the mesophilic range [4]. Hence, thermophilic fungi which are capable of secreting high yields of thermostable enzymes have gained considerable interest. One such thermophilic fungus *R. emersonii* has been reported to contain several lignocelluloytic enzymes including endo-1,4-β-glucanase, β-glucosidase, α-galactosidase, xylanase and amylases [3, 5, 6], thus possessing great industrial and biotechnological potential.

In filamentous fungi, genetic engineering can be an effective method for elucidating gene function, while also increasing production levels and minimizing unwanted byproducts [7, 8]. It is, however, very difficult to achieve homologous integration with classical genetic methods, due to the low efficiency. Lately, CRISPR/Cas (clustered regularly interspaced short palindromic repeats) technology has emerged as a cutting-edge gene editing tool to overcome filamentous fungi's low homologous integration frequency [9]. In brief, this system comprised of single guide RNA (sgRNA) and an endonuclease Cas9 enzyme which makes double-stranded break (DSB) in the specific target DNA region [10, 11]. Single guide RNA (sgRNA) consists of crRNA (CRISPR-derived RNA) that recognizes target DNA and tracer RNA (trans-activating CRISPR RNA) which interacts with Cas9 nuclease protein [12]. In order to complement target-DNA binding and make DSB, Cas9 requires the presence of a well-defined short protospacer adjacent motif (PAM) sequence (NGG) situated exactly adjacent to the non-target DNA strand. Cas9-mediated DSB is followed by either the most frequent, error-prone, non-homologous end joining (NHEJ) repair mechanism which may cause mutations, insertion or deletions in target sequence or a high-fidelity homology-directed repair (HDR) route, which needs an exogenous donor DNA or homologous DNA template for repair to make specific changes to the genome [9].

Due to high efficiency and multi-gene editing capability, CRISPR/Cas9 system has been used in various filamentous fungi including *Aspergillus oryzae* [13], *Trichoderma reesei* [14], *Aspergillus niger* [15], *Penicillium chrysogenum* [16], *Neurospora crassa* [17] and *Fusarium proliferatum* [18]. However, only a few papers have described the development and application of the CRISPR/Cas9 system in thermophilic fungus cellulase production. Recently, Liu et al. [2, 19] reported a CRISPR/Cas9 method for gene editing in thermophilic fungus *M. thermophila*. In another report, three cytosine base editors (CBEs; Mtevo-BE4max, MtGAM-BE4max, and Mtevo-CDA1) in *M. thermophila* were used for gene inactivation by precisely changing three codons (CAA, CAG, and CGA) into stop codons without the production of DSBs [20]. In *H. insolens,* a hybrid 5S rRNA–tRNA Gly promoter based CRISPR/Cas9 based disruption of pigment synthesis gene *pks* and the transcription factor *xyr1* gene was developed [21], demonstrating the significance of CRISPR technology in genetic engineering of thermophilic fungi.

The available carbon supply coordinately controls the major cellulase and hemicellulase genes, which in turn activates a number of metabolic genes and transcription factors [22]. Transcriptional activators (Xyr1/XlnR, ACE II, LAE1, BglR) and repressors (Cre1/CreA, ACE1, RCEI) work together to control the transcription of lignocellulolytic enzyme genes [23, 24]. Several fungal species have been shown to increase lignocellulolytic enzyme transcription and subsequent enzyme production by overexpressing transcriptional activators and disrupting transcriptional repressors [7, 24]. Previously in our lab, using a rigorous strain breeding and multi-omics tools, we created a hyper-lignocellulolytic *R. emersonii* strain (M36) [25]. The further development of cellulase production and strain improvement in *R. emersonii*, however, is constrained by the absence of a well-established genome editing methods. Additionally, we believe that transcription factors play a significant role in lignocellulose degradation and understanding their role is crucial for genetically manipulating *R. emersonii* to enhance its production capacity. Hence, in the current study, CRISPR/Cas9 based protocol for genetic transformation was standardized and this approach was successfully employed for the precise disruption of transcriptional repressor gene *ACE1* in *R. emersonii*. The goal of this research was to determine if *ACE1* gene disruption would further enhance enzyme production in *R. emersonii* (M36). Our findings suggest that *ACE1* gene negatively regulates the expression of cellulase and xylanase genes and is a potential target gene in strain engineering to increase the ability of *R. emersonii* to produce lignocellulolytic enzymes. This technique demonstrated excellent potential for targeted genome editing in *R. emersonii* and set the groundwork for functional genomics and the development of mutant strains with enhanced cellulase production.

## Results and discussion

### CRISPR/Cas9 mediated gene disruption in thermophilic *R. emersonii*

In order to develop a reliable CRISPR/Cas9 system**,** different parameters including antibiotic concentration, protoplast preparations and transformation protocol were first standardized (data not shown). Optimization of antibiotic was done by growing fungus on different concentrations of hygromycin (25–200 µg/mL) containing plates. The progenitor strain of *R. emersonii* (M36) showed scanty growth on 100 µg/mL hygromycin and complete growth inhibition was observed in the medium supplemented with 150 and 200 µg/mL of hygromycin (Additional file 1: Fig. S1). Therefore, for further experimentation 200 µg/mL concentration of hygromycin was selected.

**Fig. 1:**
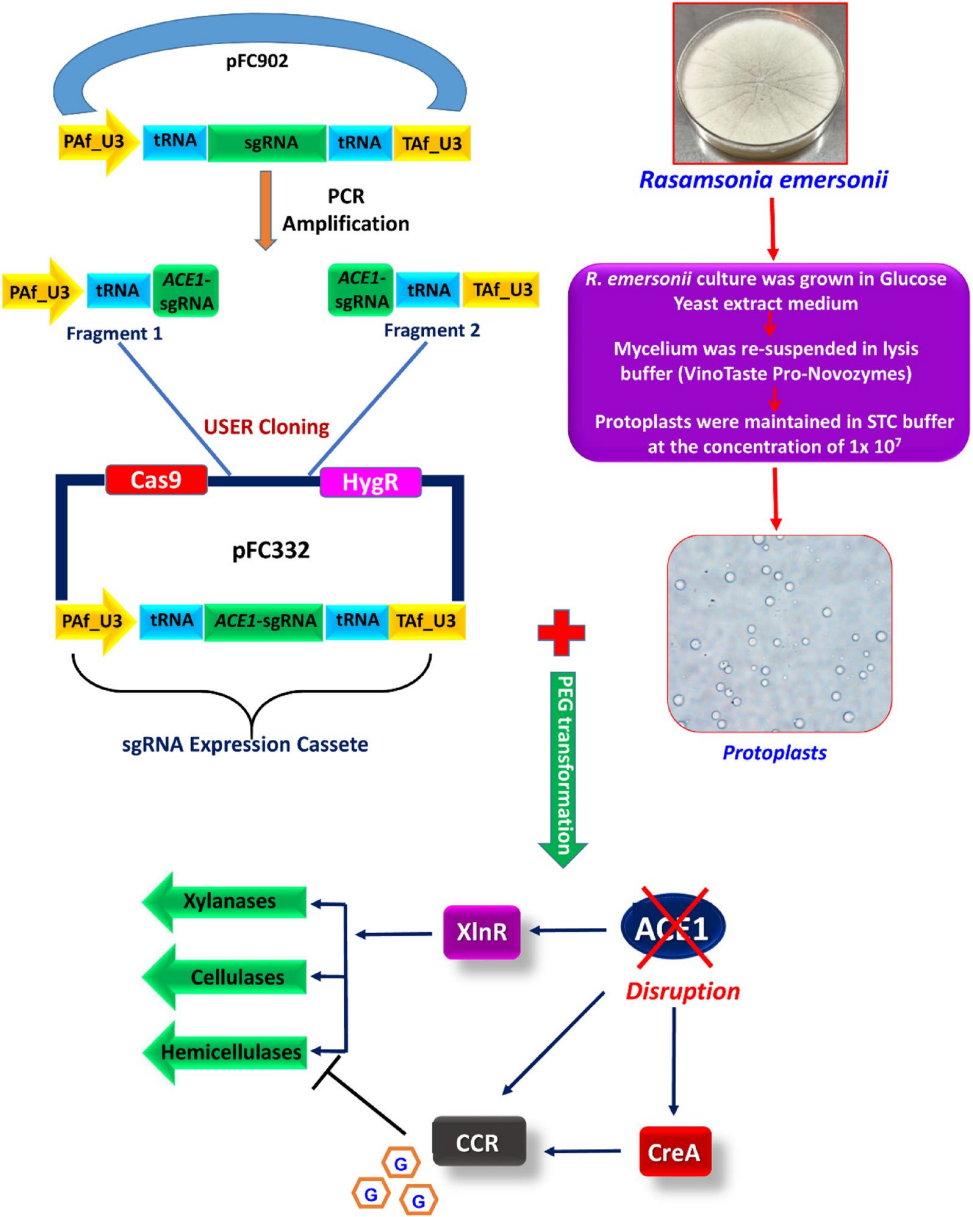
Schematic illustration depicting the CRISPR/Cas9-mediated gene editing of *ACE1* genes in *R. emersonii*. The figure illustrates the cloning strategy of *ACE1*-sgRNA, protoplast transformation, and highlights the effect of gene disruption on enhanced production of lignocellulolytic enzymes. The activation events are represented by arrows (blue) while inhibition process is represented by bar (black). CCR, carbon catabolite repression; G, glucose molecules

In the present work, one of the negative transcription regulators (ACE1) which has been reported to play critical role in suppression of cellulase and xylanase expression in *Trichoderma reesei* was targeted [26]. We transformed *R. emersonii* protoplasts with single plasmid construct (pFC322) harboring a Cas9 expressing cassette, *ACE1*-sgRNA expression cassette and hygromycin selection gene (Fig. 1). The 2187 bp *ACE1* gene of *R. emersonii* encodes a 729 amino acid long transcription factor ACE1 that showed 58.82, 54.81 and 52.94% sequence identity with *ACE1* of *Aspergillus lentulus, A. fumigatus* and *Talaromyces islandicus*, respectively (Additional file 1: Fig. S2). Efficient expression of sgRNA is strongly dependent on the promoter used; hence screening of different polymerase III promoters has been reported by Nodvig et al. [27]. In the current work, a polymerase III U3 based promoter*,* which showed the highest transformation efficiency, was used to derive the expression of *ACE1*-sgRNA.

**Fig. 2:**
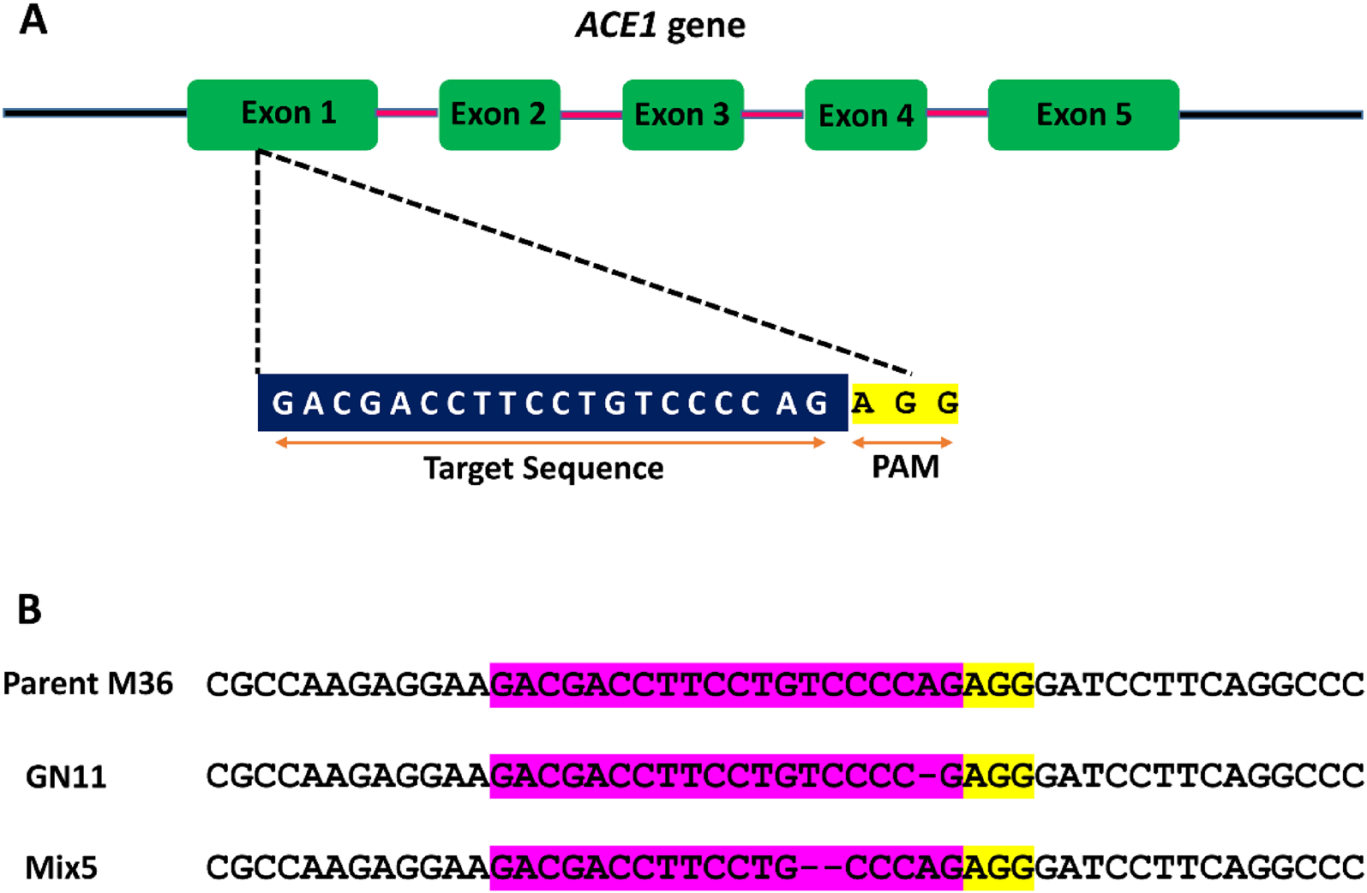
Verification of *ACE1* disruption in selected transformants **A** Target sequence (sgRNA) highlighted in blue from first exon of *ACE1* gene **B** Sequence alignment of the parental strain M36 and ∆*ACE1* mutants (GN11 and Mix5), sgRNA is highlighted in purple. Yellow letters depict the protospacer adjacent motif (PAM)

To prepare the sgRNA, Exon 1 of the *ACE1* gene was specifically chosen as the target region (Fig. 2A). Following transformation, 12 colonies were observed on the selection plates, and individual colonies were further transferred to hygromycin selection plates for the purification (Additional file 1: Fig. S3). After two rounds of sub-culturing, these colonies were transferred to hygromycin deficient media plates to cure the plasmid. Genomic DNA was isolated from these mutant strains followed by PCR amplification of *ACE1* gene fragments and sequencing. Two mutants (Mix5 and GN11) showed deletion of nucleotides preceding the PAM sequence indicating successful gene editing (Fig. 2B). Similarly, during CRISPR/Cas mediated gene editing of the thermophilic fungus *H. insolens*, Fan and coworkers [21] have also reported frame-shift mutations upstream of the PAM site involving either a single nucleotide deletion or insertion.

### Editing of *ACE1* led to increased cellulase production in *R. emersonii*

The expression of cellulase and hemicellulase is regulated by several genes, including the activators ACE3 [28], XyrI/XlnR [29], BglR [19], the carbon catabolite repressor CreI [30] and the repressor ACE1 [31]. Previously, researchers have carried out conventional genetic manipulation of these transcriptional regulators to enhance cellulase synthesis in fungi [32–35]. However, the regulatory role of ACE1 on fungal growth and cellulase production in thermophilic fungus *R. emersonii* remains unknown. To study the effect of CRISPR/Cas9-mediated gene disruption, purified transformants along with parental strain M36 strain were further screened for cellulase and hemicellulase production (Table 1). Among the different purified isolates, two mutants (GN11 and Mix5) exhibited significantly enhanced production of lignocellulolytic enzymes when compared to parent strain *R. emersonii* M36. The mutant strains (Mix5 and GN11) demonstrated an increase in endoglucanase activity of 11.72 and 16.39%, respectively, as compared to the parent strain M36. Similarly, in comparison to M36, 14.51 and 21.06% higher activity of xylanase was recorded in Mix5 and GN11 mutants, respectively. In comparison to M36 strain, GN11 transformant showed 38.06 and 23.77% increased cellobiohydrolase (CBHI) and *β*-glucosidase activity, respectively. Previously, Wang et al. [31], reported an increase in the synthesis of cellulase and xylanase enzymes in *ACE1* gene silenced strain of *Trichoderma koningii* developed by RNA interference approach. Similarly, cellulase and xylanase activity were boosted further by constitutive expression of *xyr1* and down-regulation of *ACE1 i*n *T. reesei* RUT C30 [36]. Furthermore, the comparative SDS-PAGE analysis of M36 and *∆ACE1* mutants (GN11 and Mix5) demonstrated elevated protein levels in GN11 and Mix5 when compared to M36 (Additional file 1: Fig. S4).

**Table 1:** Screening of gene edited transformants for cellulase and xylanase enzyme activities

Activity against different substrates (Units/ml)					
Mutants	Endoglucanase^a^	Cellobiohydrolase (CBHI)^b^	β-glucosidase^c^	Fpase^d^	Xylanase^e^
M36	**446.88 ± 7.12**	**11.11 ± 0.55**	**53.24 ± 1.12**	**4.02 ± 1.62**	**306.27 ± 3.27**
GN1	403.87 ± 5.18	11.13 ± 0.51	52.81 ± 1.67	3.60 ± 1.11	303.13 ± 5.44
GN2	470.40 ± 6.25	10.99 ± 1.10	44.66 ± 0.99	4.02 ± .89	281.80 ± 3.66
GN3	457.63 ± 5.14	10.30 ± .087	51.94 ± 0.58	4.00 ± .75	284.34 ± 4.12
GN4	455.61 ± 6.16	10.42 ± .043	50.21 ± 0.88	3.88 ± 1.44	274.95 ± 4.68
GN6	444.19 ± 6.54	10.26 ± 1.02	45.35 ± 1.16	4.01 ± 1.61	295.56 ± 5.99
GN11	**520.12 ± 3.16**	**15.34 ± 0.94**	**65.90 ± 2.08**	**4.46 ± 0.79**	**370.78 ± 3.58**
Mix2	461.66 ± 8.34	10.40 ± 1.43	50.21 ± 3.32	3.67 ± .92	308.14 ± 4.46
Mix3	424.70 ± 5.78	10.42 ± 0.45	45.09 ± 2.66	3.98 ± .43	291.63 ± 3.14
Mix5	**499.29 ± 4.89**	**14.11 ± 0.17**	**60.79 ± 1.94**	**4.35 ± 0.31**	**350.73 ± 3.14**
Mix6	442.84 ± 5.16	10.89 ± .053	46.39 ± 2.47	4.05 ± 1.17	279.06 ± 4.57

Data expressed as mean values (±SE), from three independent repeats

The enzyme activities were estimated against different substrates (a, Carboxymethyl cellulose (CMC); b, p-nitrophenyl-b-D-cellobioside (pNPC); c, p-nitrophenyl-b-D-glucoside (pNPG); d, filter paper, e, birchwood xylan)

### Effect of MGDs on enzyme production using batch and fed batch shake flask methods

Despite the fact that cellulose is a naturally occurring inducer of cellulase in *T. reesei*, using insoluble cellulose poses technical issues related to heterogeneous culture conditions as well as proper phase mixing, which impacts the cellulase synthesis [37]. Therefore, alternate soluble inducers mixture of glucose and disaccharide (MGDs) including sophorose, lactose, C_5_ rich acid hydrolysates have been suggested to produce copious amounts of cellulase using fed batch mode of fermentation [38, 39]. Among these, sophorose is very expensive and therefore in the present work, a low-cost cellulase overproduction mixture was prepared from glucose through *β*-glucosidase mediated transglycosylation reaction resulting in mixture of oligosaccharides sophorose, cellobiose and gentiobiose [40, 41]. The batch and fed batch flask cultures was employed in order to determine the impact of glucose/MGDs as carbon source feed on cellulase and xylanase production in the gene edited *∆ACE1* (GN11) and parental strain M36.

**Fig. 3:**
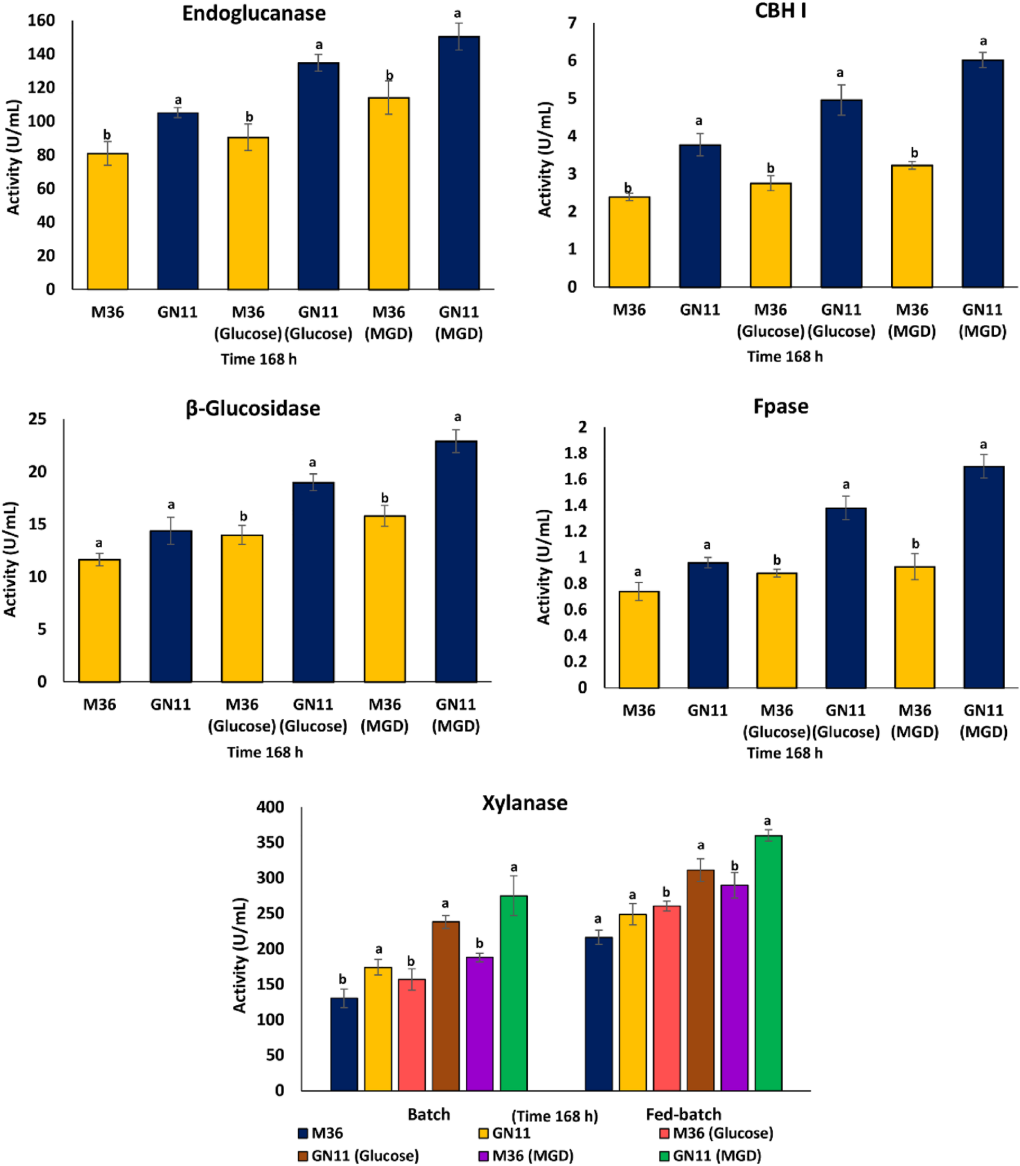
The production profile of enzymes by *R. emersonii* wild (M36), and mutant strain (GN11) under batch conditions. Values are mean of three replicates and error bars represent SD. Different letters **a**, **b** within samples are significantly different from each other (paired t-test, p ≤ 0.05)

During batch culture experiment, where the production medium was supplemented either with glucose or MGD, significant improvement in cellulase production titers were observed in strain GN11 when compared to parent strain. However, the effect was much more pronounced in presence of MGD as carbon source that comprised of 263 g/L glucose, 52.6 g/L gentiobiose, 8.7 g/L cellobiose and 14.9 g/L sophorose (Additional file 1: Table S1). Without any supplementation, GN11 showed 29.94, 33.68, 57.74, 23.59 and 29.72% enhanced activities of endoglucanase, xylanase, CBHI, *β*-glucosidase, and FPase enzymes, respectively, in comparison to M36 (Fig. 3). The addition of 1% glucose to the medium resulted in 48.91, 51.59, 80.36, 35.76 and 56.81% increase in the endoglucanase, xylanase, CBHI, *β*-glucosidase and FPase activities, respectively, as compared to M36. Whereas, addition of 1% MGDs in GN11, increased the endoglucanase, xylanase, CBHI, *β*-glucosidase and FPase activities to 31.75, 46.27, 86.37, 44.93 and 82.79%, respectively, in comparison to M36 (Fig. 3). These results were similar to Li et al. [40], who also reported a significant induction of cellulases during culturing *T. reesei* in response to the inducers (glucose, gentiobiose, cellobiose, sophorose).

**Fig. 4:**
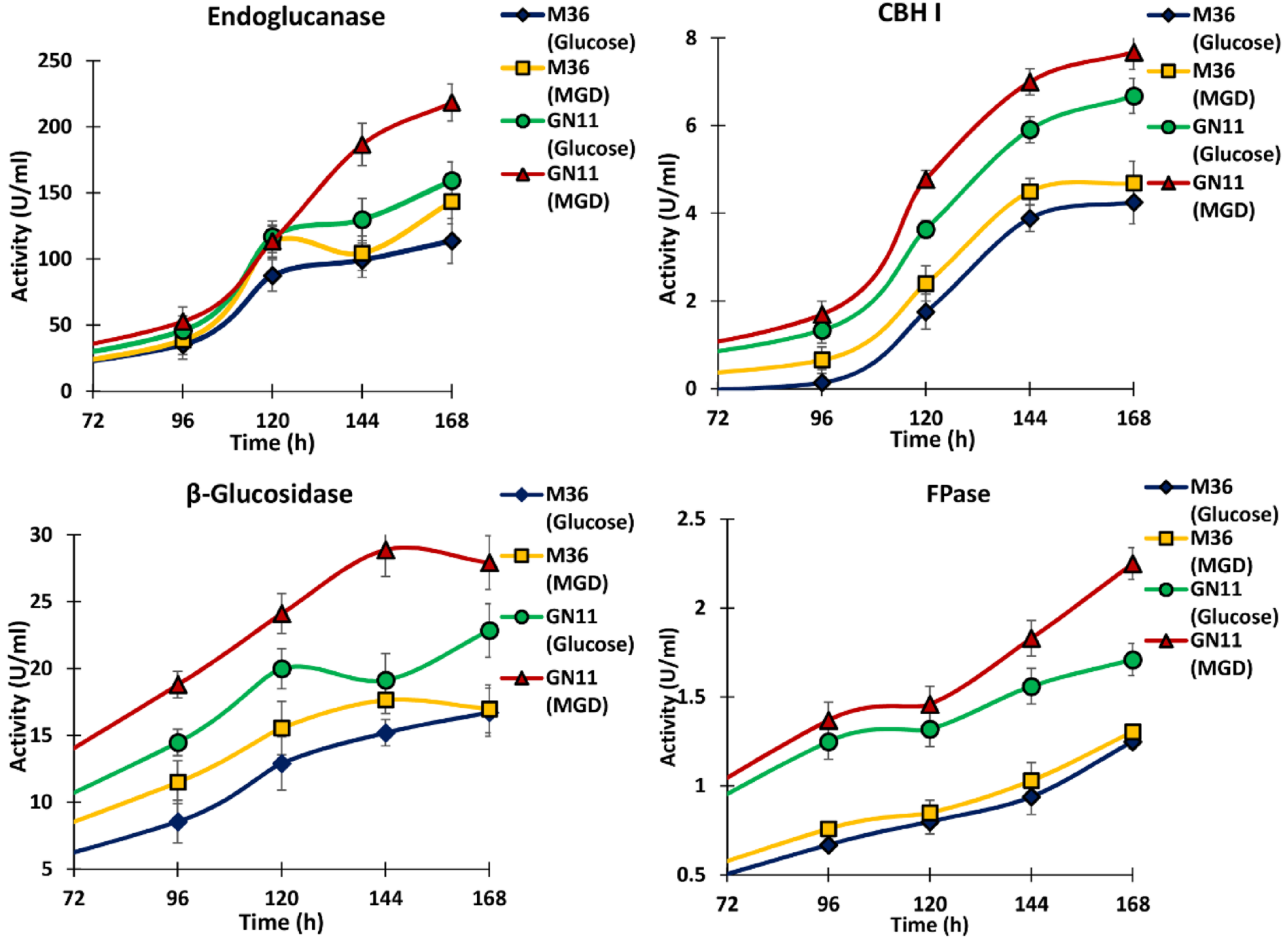
The production profile of enzymes by *R. emersonii* wild (M36), and mutant strain (GN11) using fed batch shake flask culture method. Values are mean of three replicates and error bars represent SD

The recorded lignocellulytic enzyme titers during fed batch were improved when compared to levels observed during batch e.g., the strain GN11 when step fed with glucose resulted in 40.05, 56.80, 36.58, 36.8 and 19.46%, increase in the titers of endoglucanase, CBHI, *β*-glucosidase FPase and xylanase activities, respectively, when compared to glucose fed culture of M36 (Fig. 4). When compared to M36, feeding of MGDs during culturing of GN11 lead to even higher activities of endoglucanase (52.23%), CBHI (63.75%), β-glucosidase (64.42%) and FPase (72.41%), xylanase (24.16%).

The observed enzyme titres of endoglucanase, *β* glucosidase, CBHI, FPase and xylanase during MGD fed batch culture of GN11 were 218.4, 27.9, 7.68, 2.25 and 359 units/ml when compared to 150.38, 22.9, 6.02, 1.7 and 275 units/ml, respectively, during batch cultivation of GN11 supplemented with MGD. These findings further highlight the potential of the *∆ACE1* strain, which was able to confer a constitutive phenotype to the mutant GN11. Thus, it can be exploited for the fed batch production of cellulases, as observed in genetically modified strains of *T. reesei* CCL847 and TR3002 [39].

### Effects of *ACE1* gene disruption on transcript levels of cellulase genes

**Fig. 5:**
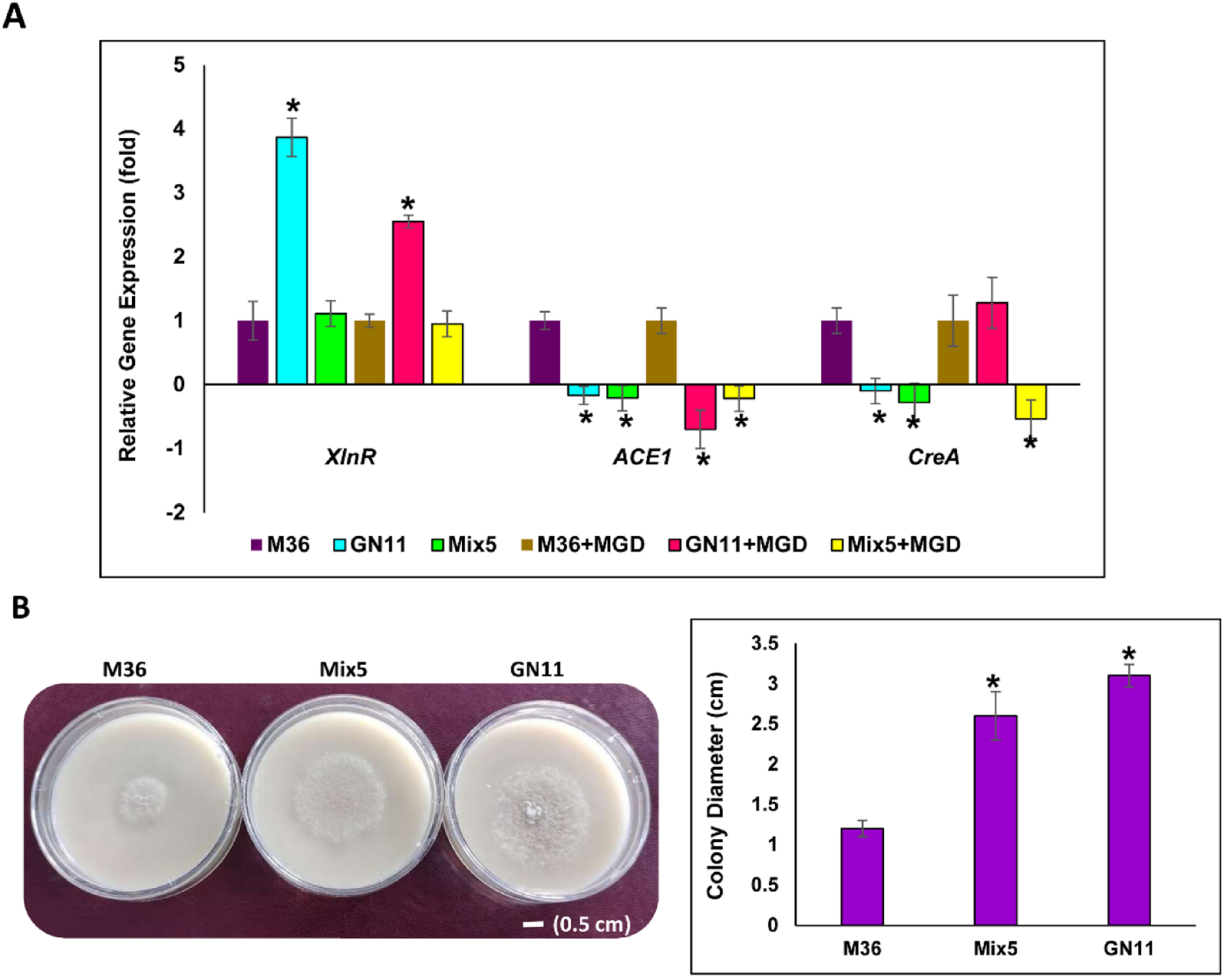
Relative gene expression analysis of transcription factors **A**. Relative expression levels were calculated using 2 ^−ΔΔCt^ method. Bars marked by asterisks in each group differ significantly from parental strain M36 and M36 + MGD (Student’s t test, p < 0.01). Error bars represent SE from three replicates. **B** Comparative growth and Radial growth (colony diameter) of M36, GN11 and Mix5 on 2-DG plates. Bars marked by asterisks in each group differ significantly from parental strain M36 (paired t-test, p ≤ 0.05)

The effect of *ACE1* gene editing on transcript level expression of regulatory genes (*CreA, XlnR* and *ACE1*) and different lignocellulolytic (CAZymes) genes in GN11 and Mix5 strain were studied. The results (Fig. 5A) showed down-regulated expression of *ACE1* and *CreA* genes which are potential negative regulators of cellulolytic enzymes production. In GN11 mutant, transcript level of *ACE1* and *CreA* was significantly down-regulated by 0.28 and 0.21 folds, respectively, when compared to parent strain M36. These results suggested the involvement of *ACE1* gene in the regulation of carbon catabolite repression in *R. emersonii*. To test this hypothesis, we checked the sensitivity of the selected mutants (GN11 and Mix5) towards 2-deoxyglucose (2-DG) which is a known toxic analogue of glucose [42]. As expected, Mix5 and GN11 showed radial growth of 2.6 cm and 3.1 cm, respectively when compared to M36 (1.2 cm) on 2-DG containing medium (Fig. 5B). So far only CreA transcription factor is known for regulating the carbon catabolite repression in filamentous fungi [43], however, we provide indirect evidence that ACE1 might also play an important role in this mechanism.

Furthermore, the expression of *xlnR* gene was 3.87 folds up-regulated in GN11 mutant as compared to M36. Our findings are consistent with Aro et al. [26] who had reported an increased expression of genes coding for xylanase and transcriptional factors *xyn1* and *xyn2,* in *ACE1* deleted transformants of *T. reesei*. Previously, three Cys2His2- zinc finger domains have been identified in *ACE1*, which bind to the *cbh1* and *xyn1* transcriptional promoter regions and thus regulate the expression of these genes [44, 45].

**Fig. 6:**
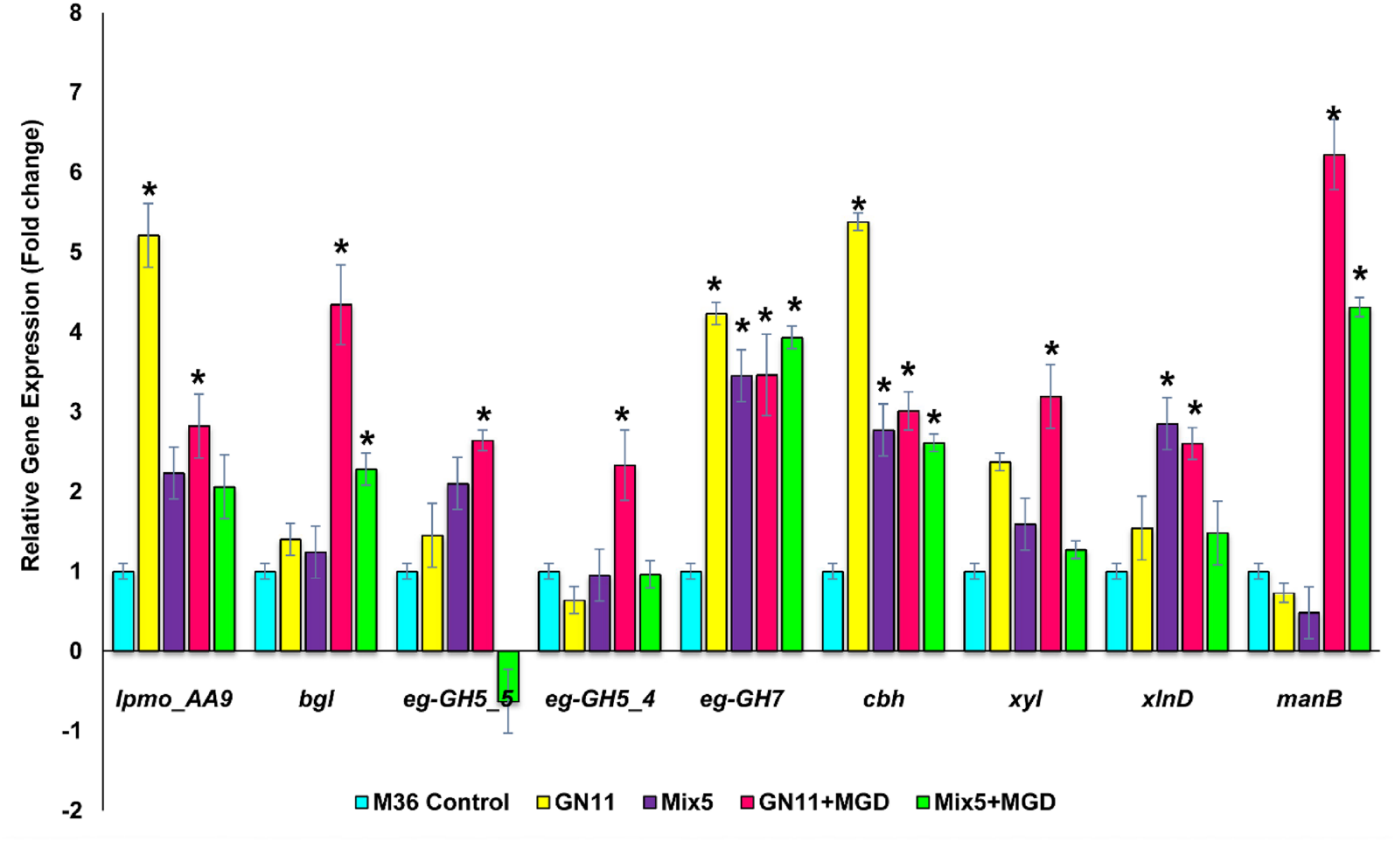
Relative gene expression analysis of various cellulase genes. Relative expression levels were calculated using 2^−ΔΔCt^ method. M36 control bar represents reference sample M36 (for GN11, Mix5) and M36 + MGD (for GN11 + MGD, Mix5 + MGD). Bars marked by asterisks in each group differ significantly from parental strain M36 (Student’s t test, p < 0.01). Error bars represent SE from three replicates

*R. emersonii* strains are known for being a source of thermostable and efficient lignocellulolytic enzymes, leading DSM to endorse it for on-site enzyme production in 2G ethanol plants [46]. In our lab, secretome analysis using Q-TOF LC/MS revealed an abundance of CAZymes [25]. In response to these revelatory insights, we conducted a comparison of the expression levels of key cellulase and auxiliary enzyme genes in *∆ACE1* strains (GN11, Mix5) and M36. The targeted genes include LPMO (*lpmo_AA9*), β-glucosidase (*bgl*), endoglucanases (*eg-GH7, eg-GH5_5, eg-GH5_4*), cellobiohydrolase C (*cbh*), xylanase (*xyl*), β-xylosidase (*xlnD*), and *α*-mannosidase (*manB*). It was found that CRISPR based gene disruption of *ACE1* resulted in enhanced expression of all the selected genes as compared to parental strain M36 (Fig. 6). In strain GN11 relative enhanced expression levels of 5.21, 5.38 and 4.23 folds were detected for *lpmo_AA9, cbh* and *eg-GH7*, respectively. Similarly, 3.45, 2.85 and 2.77 fold enhanced expression of *eg-GH7, xlnD* and *cbh* were recorded for Mix5 mutant, respectively. However, insignificant changes in the transcript levels of *manB, eg-GH5_5, bgl* and *xlnD* genes was observed in GN11 mutant. In *T. reesei*, major cellulase genes and two xylanase genes were over-expressed when *ACE1* was deleted, suggesting that *ACE1* negatively regulates the expression of these enzymes [26, 47]. In another report, Wang et al. [31] also described the enhanced transcript levels of *cbh1, egl1, bgl1,* and *xyn2* genes in *T. reesei ∆ACE1* strain. Although *ACE1* is conserved across multiple cellulolytic fungi, its role in fungal strains other than *T. reesei* has yet not been characterized. In addition, from the fed batch experiment, we observed a significant enhanced expression of cellulase enzymes using MGDs as inducers, thus we further studied the expression profile of these genes in MGDs containing medium. Addition of MGDs significantly improved the transcript levels of most of the selected genes in both GN11 and Mix5 mutants (Fig. 6). The addition of MGDs during the culturing of GN11 samples resulted in a significant upregulation of *manB* (6.22 fold), eg*-GH5_4* (2.64 fold), *bgl* (4.34 fold) and *xlnD* (2.60 fold) genes as compared to M36 grown under similar conditions (Fig. 6). The role of MGDs in global induction of different genes is an interesting observation that can be useful in production of enzyme cocktails represented with desired CAZyme components in higher amounts.

### Hydrolytic potential of *∆ACE1* strains

**Fig. 7:**
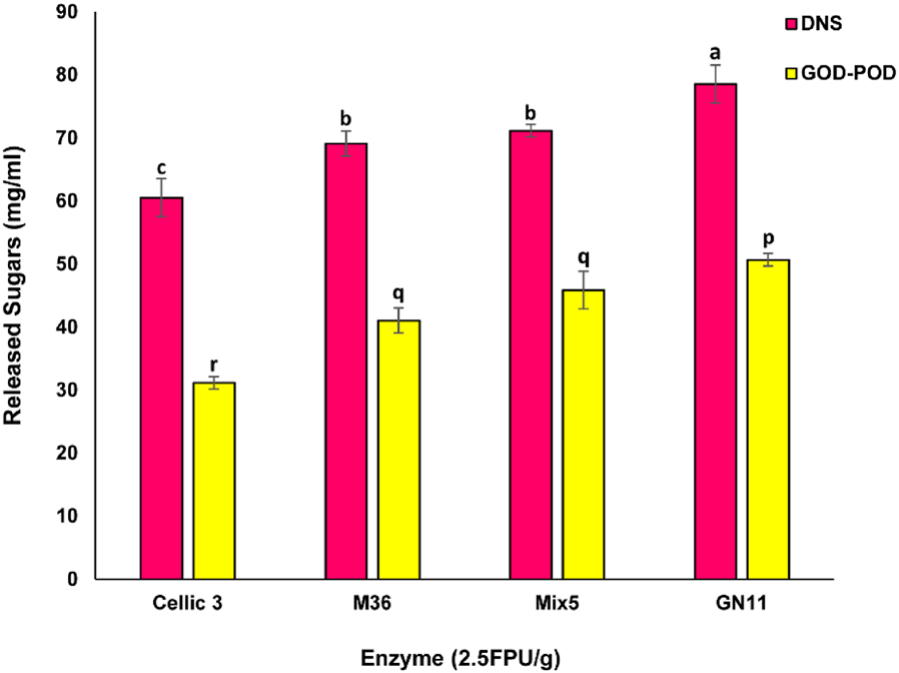
Amount of net reducing sugars (DNS) and glucose (GOD-POD) released during the saccharification of steam/acid pre-treated unwashed rice straw slurry from Praj Industries Ltd. Different letters (a, b, c and p, q, r) within samples are significantly different from each other (paired t-test, p ≤ 0.05). Values are mean of three replicates and error bars represent SD

Saccharification potential of lignocellulolytic enzyme produced by *∆ACE1* strains (GN11, Mix5) and M36 was evaluated using steam/acid pretreated unwashed rice straw slurry (15% substrate loading rate) and enzyme load of 2.5 FPU/g substrate. The performance of enzymes produced by *R. emersonii* strains performed much better when compared to industrial benchmark enzyme CelliCTec3 (Fig. 7). The results revealed that the total reducing sugars and glucose released by the cellulase from developed strain GN11 was found to be 13.63 and 23.47%, respectively, higher in comparison to the CellicCTec3. The higher hydrolytic potential of the GN11 strain may be attributed to the higher catalytic efficiency and stability of enzymes from *R. emersonii* in comparison to Cellic Ctec3, which is primarily derived from *T. reesei* strains. In addition, the *R. emersonii* secretome is represented with high levels of LPMO and swollenin [25]. As part of the study, we have observed that enzymes from *R. emersonii* strain were more resistant to the inhibitors (acetic acid, ferulic acid, furfurals) present in the slurry of acid/steam pre-treated rice straw, when compared to Cellic Ctec3 (being reported elsewhere). These findings demonstrate the potential of a strain development program that incorporates protocols such as CRISPR/Cas-mediated targeted gene editing, supported by genome-based databases and other systems biology tools, to result in the creation of substantially superior biorefineries relevant lignocellulolytic strains.

## Conclusions

This study for the first time reports the effective CRISPR/Cas9 genome editing technology for filamentous fungus *R. emersonii.* The techniques described here might also be used for other commercially significant fungi, for which there are limited tools for genetic manipulation. This approach enabled the deletion of the *ACE1* gene that resulted in up-regulation of transcriptional activator XlnR which controls the expression of cellulase genes. The ∆*ACE1* developed strains after single round of engineering showed significantly higher production of lignocellulolytic enzymes. The study exemplifies the use of CRISPR/Cas9 system to investigate the regulatory mechanisms of cellulase expression in *R. emersonii*, allowing researchers to better understand how these genes and transcriptional regulators work. This could lead to new insights into the biology and evolution of this organism, as well as potential applications in biotechnology.

## Materials and methods

### Strains and culture media

*R. emersonii* was grown at 44 °C in optimized growth media comprising of cellulose 4%, chick pea flour 2%, bactopeptone 0.75%, KH_2_PO_4_ 1%, CaCl_2_⋅2H_2_O 0.05%, ZnCl_2_ 0.034%, salt fraction 1.5% [3]. *E. coli* Top10F strain (Invitrogen, USA) was used for transformation and to maintain plasmids using 100 µg/mL ampicillin. Protoplasts regeneration media comprising of yeast extract 0.4%, K_2_HPO_4_ 0.1%, MgSO_4_⋅7H_2_O 0.05%, glucose 1.5%, sucrose 13.60% was used [25].

### Plasmid construction and cloning

To enhance the lignocellulolytic enzyme potential of this strain, one of the key potential negative regulators (*ACE1*) was selected for disruption experiment. Protospacer region of sgRNA was designed using CHOCHOP online server (https://chopchop.cbu.uib.no/). Selected protospacer and primers used are presented in Table 2. For the cloning of sgRNA, pFC332 (Number-87845) and pFC902 (Number-106904) plasmids were procured from Addgene (https://www.addgene.org/) and constructs were prepared as described by Nodvig et al. [27]. Briefly, pFC902 plasmid was used as template to generate PCR fragments which were cloned into PacI/Nt.BbvCI digested pFC332 plasmid using USER cloning (New England Biolab). Standard PCR reactions of 1X Q5U Reaction Buffer, 200 μM dNTPs, 0.5 μM forward and reverse primer, 10 ng plasmid DNA and 1U Hotstart Q5U polymerase (New England Biolab) were performed using touch down PCR with annealing temperature ranging from 67 to 56 °C. Ligated products were transformed into *E.coli* Top10F competent cells using heat shock method and positive clones were used for plasmid isolation and protoplast transformation.

**Table 2:** Details of primers used for the cloning of sgRNA and quantitative RT-PCR

Gene Name	Accession Number	Sequences (5′–3′) of paired primers	Product Size (bp)
*ACE1*	Talem1p7_004885	ATCGCCAAGAGGAAGACG/ATGATGCGCTGTCTGGA	146
*CreA*	Talem1p7_016699	AGACACATTCGGACGCATC/TGTGCTGTGCCTTGTTACT	148
*XlnR*	Talem1p7_014973	CGATCAACATGCTACGCAATC/GGTGACGATCCATCACATAGAG	129
*LPMO (AA9)*	Talem1p7_002983	GCTGTTTCTTCTACTGCTCCT/TTCGATCTCCTCCGCAATAAC	123
*β-Glucosidase A* (GH3)	Talem1p7_000240	GCTTCGACCGTATCACACTT/TAGTTGGTCACAACCCAGTTC	115
*Endoglucanse-B* (GH5_5)	Talem1p7_004873	CTCGACTATCTCGCCAACAATAC/AGAACCTGCTCATACGCAATC	125
*Endoglucanase* (GH5_4)	Talem1p7_014763	CTTCACTACCGTTCCTCTTCTG/TAGCGGTGCGGATGAAATAG	104
*Endoglucanase* (GH7)	Talem1p7_015491	GTCCCAACGAAGAGGAATGT/CCGTTCGTGACGTACTGATT	115
*CellobiohydrolaseC* (GH6)	Talem1p7_000760	ACCTGAACGTGGCGAAAT/GCGTGTCCAGCATCAAGATA	115
*Xylanase (GH10)*	Talem1p7_010333	CCAGATGTTGCGGTGTCATA/TGGCCCTTGTAATGGGTAAC	138
*β-Xylosidase* (GH3)	Talem1p7_009060	GACCCAGAGCATGTCAAGAT/ATCCTGCTGCGTGATGATAG	114
*α*-*Mannosidase* (GH47)	Talem1p7_011066	CCCATCTCTGTCACTCTCTTTG/TTATACCCGTCCCAAGCAATC	144
*EF-1*	Talem1p7_017962	CCAGGGTCCCAAGAAAGAAA/TACCCAGGCCTAGCTTCTTA	111
VS_ACE1-F-902	Talem1p7_004885	ACCTTCCUGTCCCCAGGTTTTAGAGCTAGAAATAGCAAGTTAAA	–-
VS_ACE1-R-902		AGGAAGGUCGTCTGCATCATCCGTGAATCGAAC	–-
VS_CSN438-F	–-	GGGTTTAAUGATCACATAGATGCTCGGTTGACA	–-
VS_CSN790-R	–-	GGTCTTAAUACCCTGAGAAGATAGATGTGAATGTG	–-
*ACE1*-sgRNA	–-	GACGACCTTCCTGTCCCCAG	–-

### Protoplast preparation and transformation

Freshly inoculated 7 days old *R. emersonii* culture grown on wheat flour based agar medium [3] was used to inoculate 250 mL flasks containing 50 mL of liquid medium containing 2% glucose and 2% yeast extract. After 16 h of incubation at 44 °C, mycelium was filtered through two layers of sterile Miracloth (Merck Millipore) and washed twice with sterile water. Harvested mycelium was kept in freshly prepared 10 (mg/mL) of lysing enzyme (VinoTaste Pro in 1 M sorbitol, 100 mM sodium phosphate pH 6.0) for 3 h at 28 °C with gentle shaking (50 rpm). The protoplasts were checked under microscope (Olympus BX60) and filtered through three layers of Miracloth to collect them into sterile centrifuge tubes. Protoplasts solution was centrifuged at 4000 rpm for 10 min and the pellet was re-suspended in STC (1 M sorbitol, 50 mM CaCl_2_, 10 mM Tris–HCl; pH 7.5) buffer by gentle pipetting. Protoplasts concentration was finally adjusted to 10^7^/mL and further used for PEG mediated transformation. Protoplasts aliquots (100 µL) were mixed with 5 µg of purified plasmid and incubated for 30 min in ice. After that, 1 mL of freshly prepared PEG solution (50% Polyethylene glycol 3350 in 50 mM CaCl_2_, 10 mM Tris–HCl; pH 7.5) was slowly added and further incubated at room temperature for 20 min. Finally, 2 mL of STC solution followed by 3 mL of regeneration medium was added to the protoplasts solution and incubated at 40 °C for 24 h. Next day regenerated protoplasts were mixed with slightly warm (45–50 °C) regeneration medium containing hygromycin 200 µg/mL and further incubated at 44 °C till colonies appeared (usually 4–5 days). Firstly, each resultant colony was purified by streaking onto hygromycin selection plates and later two consecutive streaking were done onto non-selective medium to lose the plasmid. Mutant isolates were used to isolate genomic DNA using DNA isolation kits (HiMedia, India) and gene editing was verified by sequencing PCR-amplified products (BioServe, Hyderabad).

### Lignocellulolytic enzymes production and enzymatic assays

The isolated *∆ACE1* mutants were screened for the lignocellulolytic enzymes production using shake flask culture technique [25]. Four stubs were taken from 1-week-old plates of mutants and the parental culture, and used to inoculate 50 ml of optimized production media in 250 ml conical flasks. The flasks were then kept in a shaker set at 250 rpm at 44 °C for 7 days. After incubation, the contents of the flasks were harvested by filtration and subsequently subjected to centrifugation at 10,000 g for 20 min. The resulting clarified extract was then assayed for lignocellulolytic activities.

Enzyme extracts of *∆ACE1* mutants and parental culture were checked for various enzymatic activities as described in our previous lab paper [3]. For endoglucanase and xylanase activities, 2% carboxymethyl cellulose (CMC) and 1% birchwood xylan substrates were mixed with suitably diluted enzyme extract and incubated at 50 °C for 10 min and 5 min, respectively. Reactions were stopped by adding 3 mL of di-nitrosalicylic acid (DNS) solution followed by boiling for 10 min [48]. The amount of sugar released was estimated by taking absorption at 540 nm (Novaspec II spectrophotometer, Pharmacia). The *β*-glucosidase and cellobiohydrolase (CBHI) activity were assayed using 3 mM para-nitrophenyl-β-D-glucopyranoside (pNPG) and p-nitrophenyl-β-cellobioside (pNPC) as respective substrates. A 100 µl reaction mix that contained 25 µl each of diluted enzyme and respective substrates (pNPG, pNPC) in sodium acetate buffer (50 mM; pH 5.0) was incubated at 50 °C for 30 min. Reactions were terminated by the addition of NaOH-glycine buffer (0.4 M, pH 10.8) and absorption was recorded at 405 nm using a plate reader (Fluostar Omega, BMG Labtech). For Total cellulase activity (FPase) measurements a filter paper strip (Whatman No. 1; 1 × 6 cm) was incubated with 1 mL of sodium acetate buffer (50 mM, pH 4.8) and suitably diluted enzyme at 50 °C for 60 min. Three milliliters of DNS was added to stop the reaction followed by boiling at 100 °C in a water bath for 10 min. The content of the tube was further diluted 12.5 times and the developed color was read at 540 nm [49].

### Effect of MGDs on enzyme production under batch and fed batch conditions

Mixture of glucose and disaccharide (MGDs) was prepared by transglycosylation of glucose with the thermophilic *β*-glucosidase purified from *R. emersonii* (expressed in *Pichia pastoris*, data will be reported elsewhere). For the production of MGDs, 50% cold sterilized glucose (5 g/10 mL of 100 mM sodium citrate buffer pH 5.0) and *β*-glucosidase (20 U/g glucose) were incubated at 60 °C for 72 h at 100 rpm [40]. Reaction was terminated by boiling in a water bath for 5 min. MGDs production was verified by HPLC using Dionex 3000 ultimate HPLC system fitted with Aminex HPX-87H column and refractive index detector (Shodex RI-101) [50]. The selected *∆ACE1* mutants and the *R. emersonii* progenitor strain (M36) were studied for fed batch and batch production of lignocellulolytic enzymes using 250 mL flasks that contained 50 mL of the culture medium (1% cellulose powder and 0.5% chick pea flour and bactopeptone 0.75%, KH_2_PO_4_ 1%, CaCl_2_⋅2H_2_O 0.05%, ZnCl_2_ 0.034%; salt fraction 1.5% and supplemented with either 1% glucose/1% MGDs. The fermentation was initiated by inoculating 50 mL culture medium contained in 250 mL Erlenmeyer flasks with 4 stubs taken from 7 days old cultures plates and incubated at 44 °C for upto 10 days. Whereas, for the fed flask mode the experiments were run for 48 h as batch and thereafter the flasks were fed with 1% glucose/1% MGDs at 24 h intervals. For profiling the production, the samples were drawn at different intervals and assayed for lignocellulolytic activities. The experiment was performed in duplicate and results were reported as mean values.

### Quantitative real time PCR (qRT-PCR) analysis of lignocellulolytic genes

For the gene expression analysis studies GN11, Mix5 and progenitor strain *R. emersonii* (M36) were grown for 7 days on optimized production medium. Mycelium was harvested and ground to fine powder using pre-cooled mortar pastel with liquid nitrogen and 100 mg of powder was used to isolate RNA (RNA isolation kit; Himedia, India). Isolated RNA was treated with DNAse (Sigma, USA) as per manufacturer details and quantified using Nanodrop (ThermoScientific, USA). Two µg of RNA was used for cDNA preparation using iScript cDNA synthesis kit (Bio-Rad, USA) and further used for qRT-PCR reactions. The selected genes included those coding for hemicellulases (xylanase_GH10 and β-xylosidase_GH3, three endoglucanases (endoglucanase_GH7, endoglucanase-B_GH5.5, endoglucanase_GH5.4), β-glucosidase A_GH3, cellobiohydrolase-C_GH6, LPMO (AA9), and *α*-mannosidase_GH47 proteins. Gene specific primers were prepared using PrimerQuest tool of IDT (https://www.idtdna.com/pages/tools/primerquest). qRT-PCR reactions were setup in 96 well plates comprised of 5 µL of SYBR Green (Bio-Rad, USA), 1 µM of forward and reverse primers and 50 ng of diluted cDNA samples. Negative controls were also included which contain sterile water instead of cDNA sample. Reactions were performed using Bio-Rad CFX96 Touch Real Time instrument (Bio-Rad, USA) under following conditions: Initial denaturation at 95 °C for 30 s, followed by 40 cycles of denaturation at 95 °C for 10 s, annealing and extension at 60 °C for 30 s and melt curve step in the range from 65 °C to 95 °C. Cycle threshold (Ct) and baseline values were selected by default instrument software and the expression analysis was performed using elongation factor (*EF-1*) as reference gene.

### Growth of Δ*ACE1* mutants in the presence of 2-deoxyglucose (2-DG)

Further to study the effect of *ACE1* inactivation on carbon catabolite repression, *ΔACE1* mutants were allowed to grow on 2-DG. Spore suspension of 10^7^ concentration was prepared and 5 µL was used to inoculate media plates supplemented with 0.8% (w/v) 2-DG (Himedia, India) and incubated at 44 °C. Colony diameter was measured after 7 days of incubation and growth on plates were photographed.

### Enzymatic hydrolysis

The hydrolytic potential of the enzyme produced by the selected strains of *R. emersonii* and the commercial cellulase blend Cellic Ctec3 were used for saccharification of unwashed acid/steam pretreated rice straw slurry obtained from Praj Industries Ltd (Pune, Maharashtra, India). Hydrolysis was performed in 15 mL glass vials containing 1 g pretreated substrate slurry and respective enzyme added at 2.50 FPU/g substrate (15% substrate loading rate; 1 g pretreated slurry was equivalent to 0.15 g on dry weight basis). The pH was adjusted to 5.0 ± 0.2 and incubated at 50 °C, 200 rpm for 72 h. After the hydrolysis glucose and total reducing sugars in the hydrolysates were estimated using GOD-POD, and DNS method, respectively [51].

### Supplementary Information

**Additional file 1: Figure S1.** Growth of *R. emersonii* on different concentration of Hygromycin. **Figure S2.** Multiple amino acid sequence alignment of ACEI from *R. emersonii*. **Figure S3.** Verification of *ACE1* disruption in selected transformants. **Figure S4.** Comparative SDS-Page analysis of M36 and mutant strains (GN11 and Mix5). Table 1: Quantitative levels of major components in MGDs.

## Data Availability

The datasets used and/or analyzed during the current study are available from the corresponding author on reasonable request.
