# Supplementary material for: CRISPR/Cas9 mediated gene editing of transcription factor ACE1 for enhanced cellulase production in thermophilic fungus Rasamsonia emersonii
Source: Fungal Biol Biotechnol. 2023 Sep 1;10:18. doi: 10.1186/s40694-023-00165-y (PMC10472679; doi:10.1186/s40694-023-00165-y)
Supplement: Supplementary file 1 — Additional file 1: Figure S1. Growth of R. emersonii on different concentration of Hygromycin. Figure S2. Multiple amino acid sequence alignment of ACEI from R. emersonii. Figure S3. Verification of ACE1 disruption in selected transformants. Figure S4. Comparative SDS-Page analysis of M36 and mutant strains (GN11 and Mix5). Table 1: Quantitative levels of major components in MGDs. [file 40694_2023_165_MOESM1_ESM.docx]

**Additional Figure S1. Growth of *R. emersonii* on different concentration of Hygromycin.**

25ug 50ug 100ug


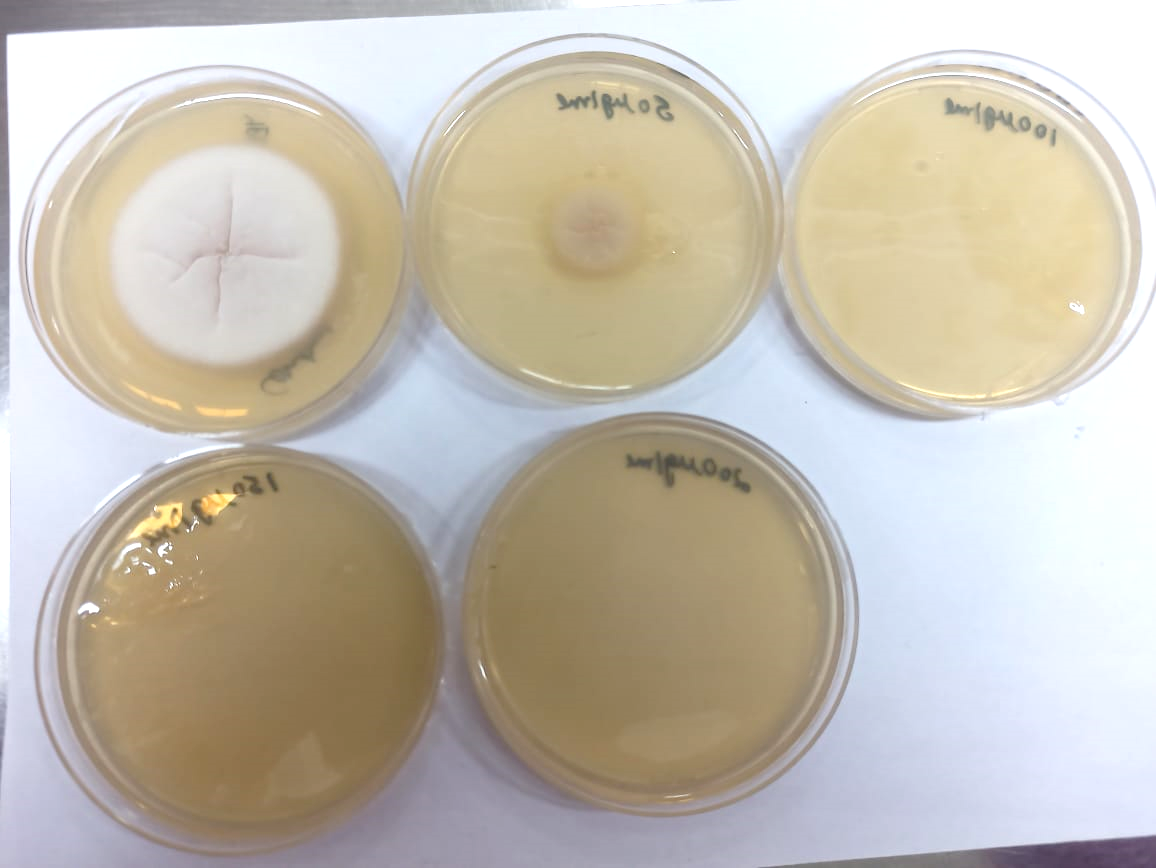


150ug 200ug

**Supplementary Figure S2. Multiple amino acid sequence alignment of ACEI from *R. emersonii*.** XP_033419838.1 (*Aspergillus lentulus*), XP_754813.1 (*Aspergillus fumigatus*), CRG88295.1 (*Talaromyces islandicus*)

.
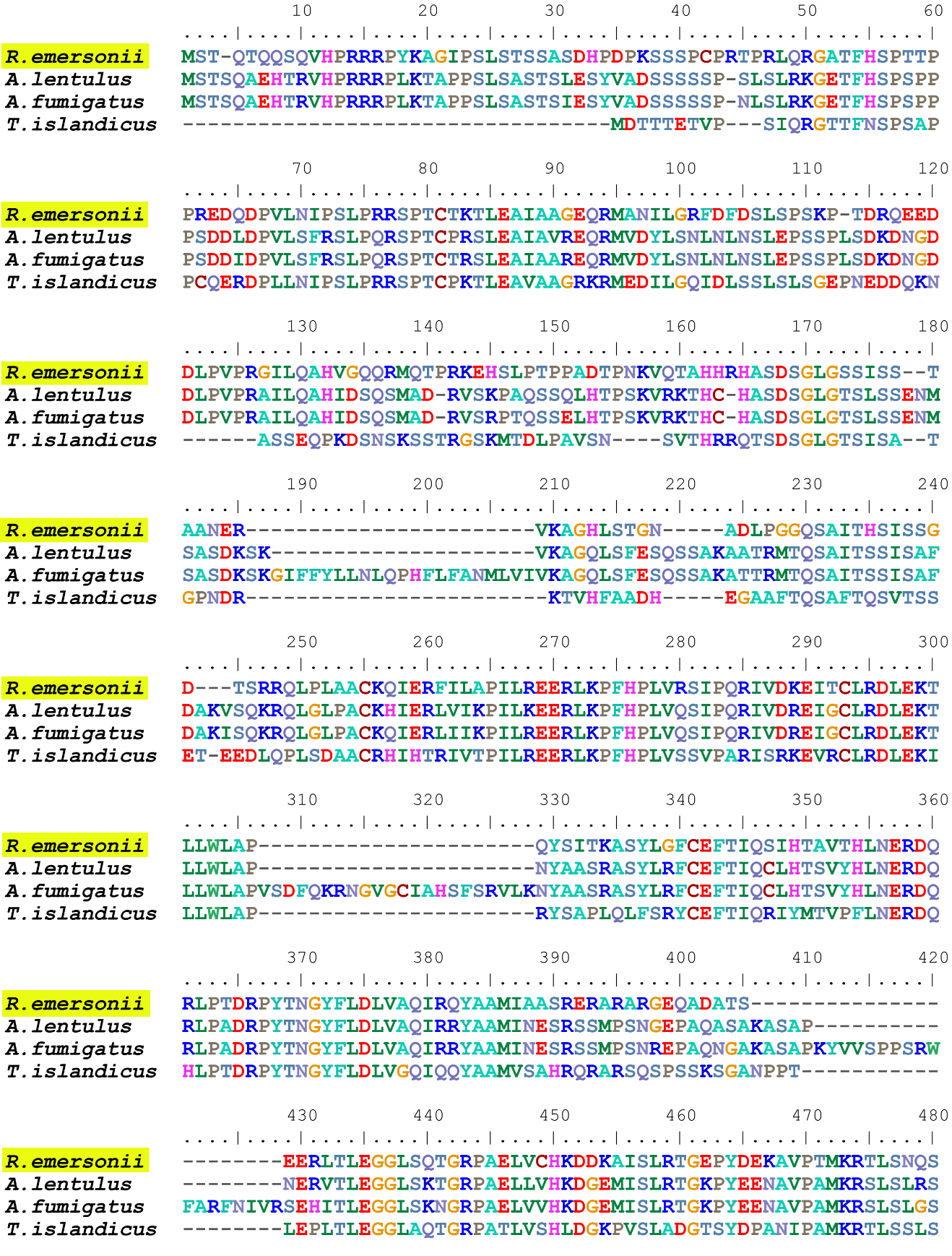


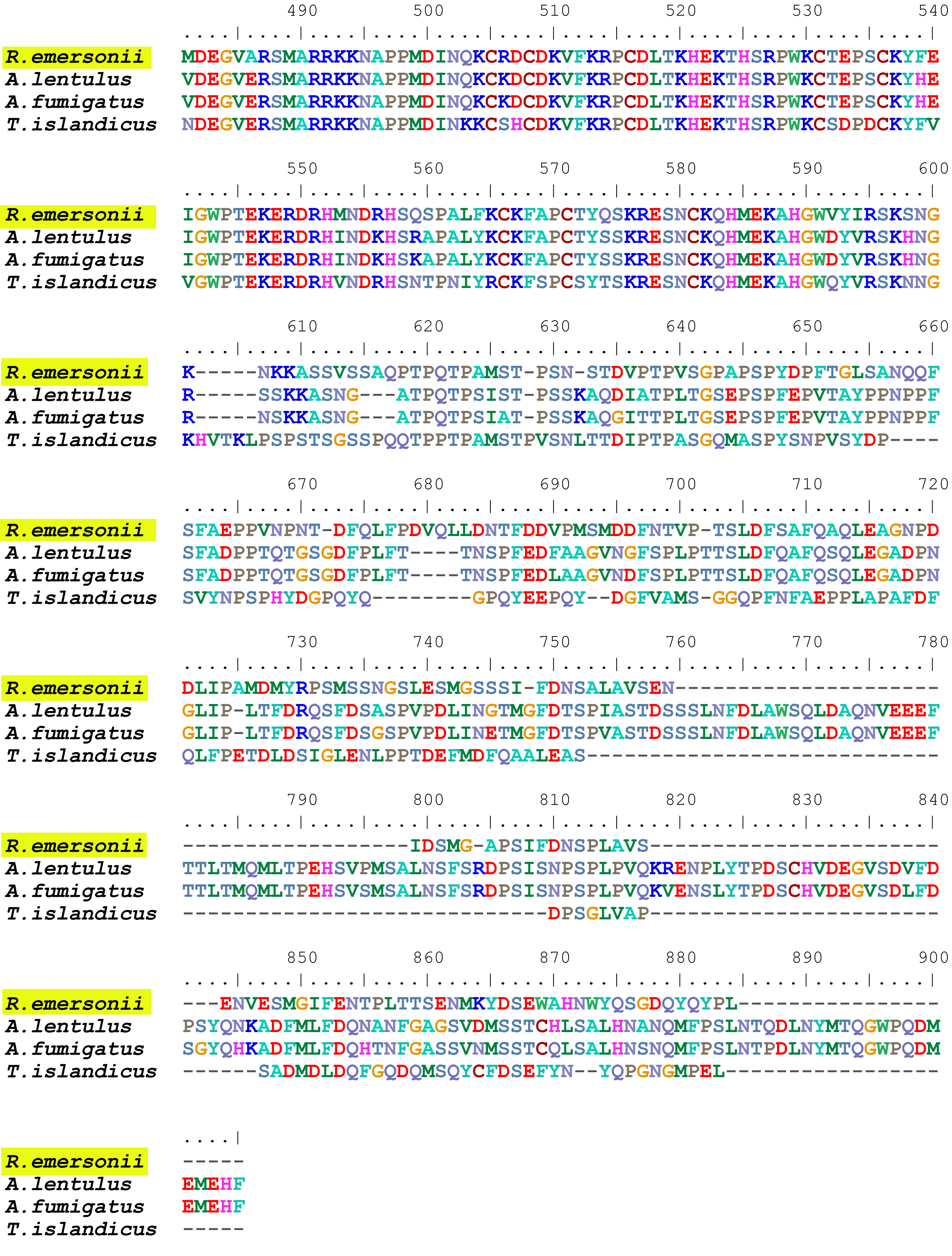


**Supplementary Figure S3. Verification of *ACE1* disruption in selected transformants (A) *ACE1* strains growing on the hygromycin selection plate (B) PCR amplified fragment of *ACE1* gene.**


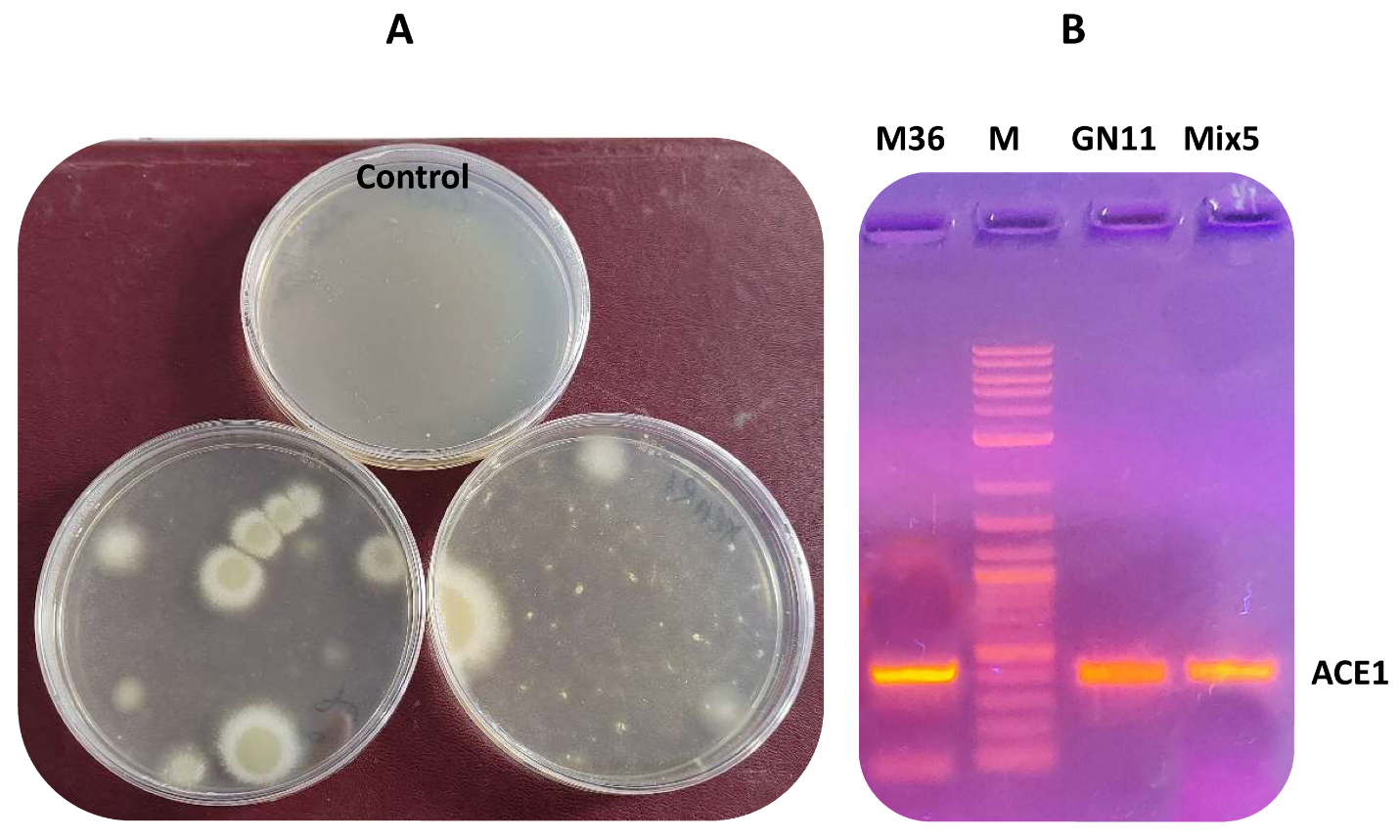


**Supplementary Figure S4. Comparative SDS-Page analysis of M36 and mutant strains (GN11 and Mix5)**

Marker M36 GN11 Mix5


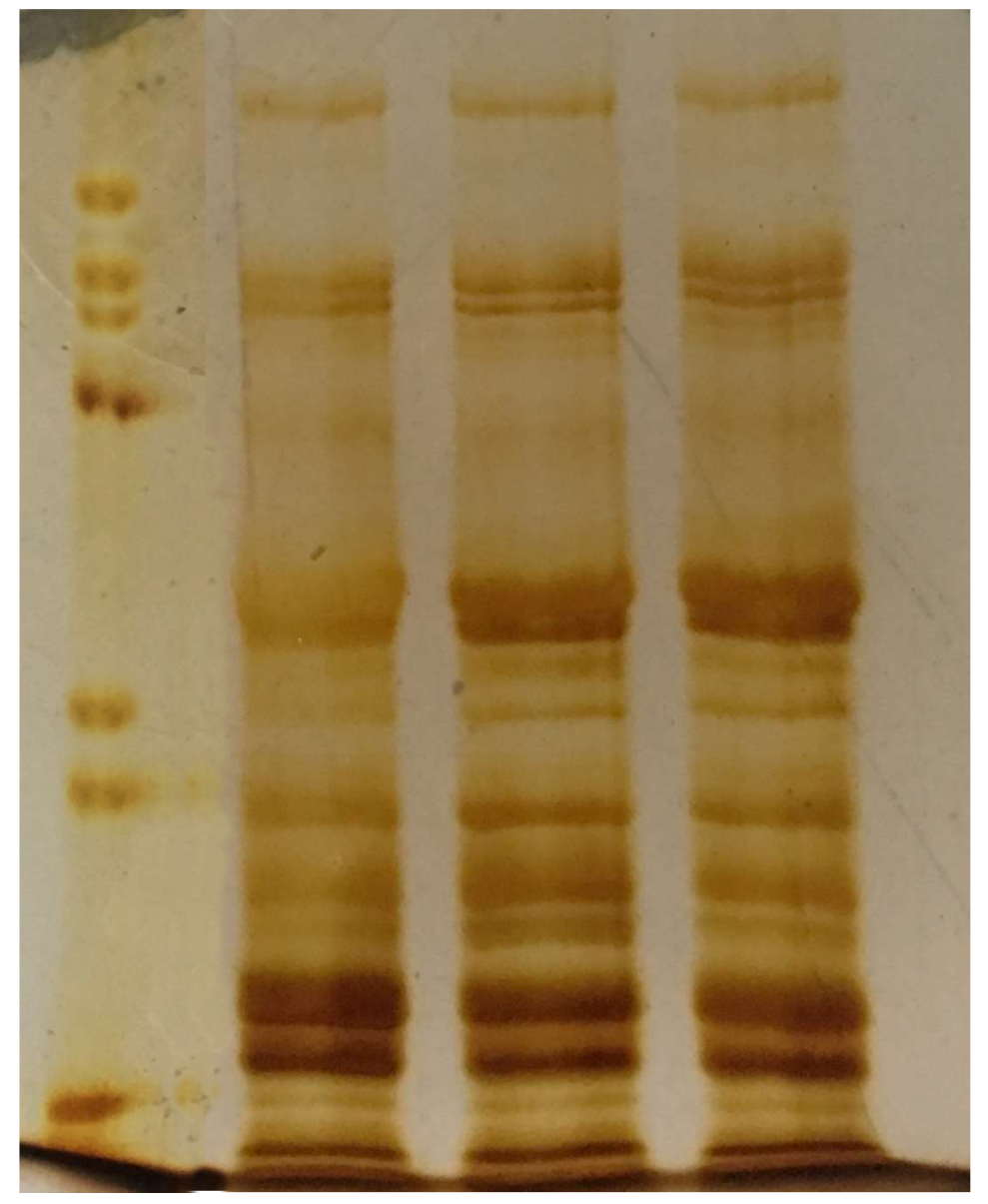


**Supplementary Table 1: Quantitative levels of major components in MGDs.**

| Glucose (g/L) | Gentiobiose (g/L) | Cellobiose (g/L) | Sophorose (g/L) |
| --- | --- | --- | --- |
| 263 ± 12.4 | 52.6 ± 3.87 | 8.7 ± 1.16 | 14.9 ± 1.66 |

The MGDs were prepared from 50% glucose through transglycosylation reaction performed at 60°C for 72 h, catalyzed by *β*-glucosidase from *R.emersonii*.
